# A phase II dose evaluation pilot feasibility randomized controlled trial of cholecalciferol in critically ill children with vitamin D deficiency (VITdAL-PICU study)

**DOI:** 10.1186/s12887-023-04205-9

**Published:** 2023-08-14

**Authors:** Katie O’Hearn, Kusum Menon, Hope A. Weiler, Karin Amrein, Dean Fergusson, Anna Gunz, Raul Bustos, Roberto Campos, Valentina Catalan, Siegfried Roedl, Anne Tsampalieros, Nick Barrowman, Pavel Geier, Matthew Henderson, Ali Khamessan, Margaret L. Lawson, Lauralyn McIntyre, Stephanie Redpath, Glenville Jones, Martin Kaufmann, Dayre McNally, the Canadian Critical Care Trials Group

**Affiliations:** 1 Research Institute, Children’s Hospital of Eastern Ontario, 401 Smyth Road, Ottawa, ON K1H 8L1 Canada; 2 Department of Pediatrics, Faculty of Medicine, University of Ottawa, Children’s Hospital of Eastern Ontario, Ottawa, Canada; 3 School of Human Nutrition, Faculty of Agricultural and Environmental Sciences, McGill University, Montreal, Canada; 4 Division of Endocrinology and Diabetology, Department of Internal Medicine, Medical University of Graz, Graz, Austria; 5 Faculty of Medicine, University of Ottawa, Ottawa, Canada; 6 Department of Paediatrics, Schulich School of Medicine & Dentistry, Western University, London, ON N6A 5W9 Canada; 7 Child Health Research Institute, London, ON N6A 5W9 Canada; 8 Clínica Sanatorio Alemán, Unidad de Cuidados Intensivos Pediátricos, Concepción, Chile; 9 Facultad de Medicine Y Ciencia, UCI Pediátrica Hospital Guillermo Grant Benavente Concepción, Universidad San Sebastián, Concepción, Chile; 10 Department of Paediatrics and Adolescent Medicine, Joint Facilities, Medical University of Graz, Graz, Austria; 11 Clinical Research Unit, Children’s Hospital of Eastern Ontario, 401 Smyth Road, Ottawa, ON K1H 8L1 Canada; 12 Department of Pathology and Laboratory Medicine, Faculty of Medicine, University of Ottawa, Children’s Hospital of Eastern Ontario, Newborn Screening Ontario, Ottawa, Canada; 13 Euro-Pharm International Canada Inc, Montreal, Canada; 14 Division of Endocrinology, Department of Pediatrics, Children’s Hospital of Eastern Ontario, Ottawa, Canada; 15 Department of Medicine (Division of Critical Care), Ottawa Hospital Research Institute (OHRI), University of Ottawa, Ottawa, Canada; 16 Department of Biomedical and Molecular Sciences, Queen’s University, Kingston, Canada

**Keywords:** Vitamin D, Pediatrics, Critical care, Feasibility, Randomized controlled trial, Vitamin D deficiency

## Abstract

**Background:**

Vitamin D deficiency (VDD) is highly prevalent in the pediatric intensive care unit (ICU) and associated with worse clinical course. Trials in adult ICU demonstrate rapid restoration of vitamin D status using an enteral loading dose is safe and may improve outcomes. There have been no published trials of rapid normalization of VDD in the pediatric ICU.

**Methods:**

We conducted a multicenter placebo-controlled phase II pilot feasibility randomized clinical trial from 2016 to 2017. We randomized 67 critically ill children with VDD from ICUs in Canada, Chile and Austria using a 2:1 randomization ratio to receive a loading dose of enteral cholecalciferol (10,000 IU/kg, maximum of 400,000 IU) or placebo. Participants, care givers, and outcomes assessors were blinded. The primary objective was to determine whether the loading dose normalized vitamin D status (25(OH)D > 75 nmol/L). Secondary objectives were to evaluate for adverse events and assess the feasibility of a phase III trial.

**Results:**

Of 67 randomized participants, one was withdrawn and seven received more than one dose of cholecalciferol before the protocol was amended to a single loading dose, leaving 59 participants in the primary analyses (40 treatment, 19 placebo). Thirty-one/38 (81.6%) participants in the treatment arm achieved a plasma 25(OH)D concentration > 75 nmol/L versus 1/18 (5.6%) the placebo arm. The mean 25(OH)D concentration in the treatment arm was 125.9 nmol/L (SD 63.4). There was no evidence of vitamin D toxicity and no major drug or safety protocol violations. The accrual rate was 3.4 patients/month, supporting feasibility of a larger trial. A day 7 blood sample was collected for 84% of patients. A survey administered to 40 participating families showed that health-related quality of life (HRQL) was the most important outcome for families for the main trial (30, 75%).

**Conclusions:**

A single 10,000 IU/kg dose can rapidly and safely normalize plasma 25(OH)D concentrations in critically ill children with VDD, but with significant variability in 25(OH)D concentrations. We established that a phase III multicentre trial is feasible. Using an outcome collected after hospital discharge (HRQL) will require strategies to minimize loss-to-follow-up.

Trial Registration.

Clinicaltrials.gov NCT02452762 Registered 25/05/2015.

**Supplementary Information:**

The online version contains supplementary material available at 10.1186/s12887-023-04205-9.

## Background

Critical illness requiring ICU admission occurs in upwards of 10,000 children each year in Canada [1]. In addition to death, these children are at high risk for new morbidity, prolonged periods of rehabilitation and significant chronic disease [2–5]. Vitamin D deficiency (VDD) (defined as a 25-hydroxyvitamin D concentration of < 50 nmol/L) is highly prevalent in pediatric intensive care units (PICU) around the globe, with rates ranging from 30 to 80% [6–12]. Further, synthesis of data from multiple observational studies have demonstrated an association between lower vitamin D levels and organ dysfunction, health resource utilization and mortality in both critically ill children and adults [6, 8–10, 12–27]. Based on the importance of adequate vitamin D status for the proper function of multiple organ systems central to critical illness pathophysiology, vitamin D supplementation has been hypothesized as a potentially simple, inexpensive, and safe intervention for improving outcomes in critically ill children [28–30]. A role for vitamin D in critical illness has biological plausibility as there are multiple mechanisms through which deficiency could contribute to organ dysfunction, including: (i) exacerbation of critical illness related hypocalcemia [31–33]; (ii) cardiovascular dysfunction indirectly through low body calcium stores and directly through vitamin D receptors (VDR) present on myocytes and endothelial cells; (iii) immune dysregulation through functional VDR present on all major immune cell types [34–36]; (iv) through the role of vitamin D signaling in innate immunity [37–39]; iv) exacerbation of critical illness polyneuropathy and muscular weakness [40–43].

To date, there have been no interventional trials in the PICU setting and the adult literature is inconclusive with some but not all demonstrating that an enteral loading dose of cholecalciferol may reduce length of stay [44], prevent mortality [45] and improve long-term functional outcomes [45–47]. A large adult phase III trial is presently underway in Europe, and will hopefully provide a clearer answer to this question [48]. Findings from adult trials, however, cannot be extrapolated to pediatrics given differences in dosing, metabolism, co-morbidities, presenting diagnoses, and clinical outcomes [49, 50]. Consequently, to determine whether vitamin D supplementation can improve outcomes in VDD critically ill children, pediatric trials are required.

Prior to undertaking a large randomized controlled trial (RCT) in the PICU setting, it is first essential to identify a dosing strategy that can safely restore vitamin D status in a timeframe relevant for critical illness. The current standard approach to vitamin D supplementation for the general pediatric population (200 to 1,000 IU/day of cholecalciferol) can take months to restore vitamin D status in otherwise healthy children who are deficient [51]. Importantly, some studies in hospitalized and ICU patients have shown that 25(OH)D concentrations may decline during the initial days or week(s) of admission with standard supplementation [52, 53] aggravated by interventions such as blood loss, blood transfusion, cardiopulmonary bypass (CPB), extracorporeal membrane oxygenation (ECMO), and plasma exchange [7, 54, 55]. While the Institute of Medicine guidelines outline a daily age-based tolerable upper limit of 1,000 to 4,000 IU/day [56], evaluation of studies using this regimen indicates many weeks and often a month or more may be required to restore vitamin D status [51]. Loading dose therapy (vitamin D dose between 40,000 and 600,000 IU as a single administration, or divided over 2 days) [51], may represent a more efficient approach to rapidly restore vitamin D status in critically ill children. However, there have been no studies evaluating the safety and efficacy of a loading dose regimen in critically ill children.

We conducted a prospective double-blind dose evaluation phase II pilot feasibility RCT. Our primary objective was to determine whether a weight-based enteral loading dose of cholecalciferol [51] can rapidly normalize vitamin D status in critically ill children. Our secondary objectives were to: (i) evaluate whether the cholecalciferol loading dose, when compared with usual care, resulted in greater occurrence of vitamin D related adverse events, and (ii) evaluate the feasibility of a multicentre phase III trial by evaluating recruitment, protocol adherence, blinding, and seeking input from participants on a patient oriented outcome.

## Methods

### Study design

This trial is reported according to the Consolidated Standards of Reporting Trials (CONSORT) statement extension for pilot and feasibility trials (see checklist, Additional file 1) [57, 58]. We conducted an international, multi-center, double-blind, phase II dose evaluation randomized controlled trial from January 2016 to November 2017. The study rationale, design, and protocol have been published [59]. Ethical approval was obtained from the research ethics board at each participating site. Regulatory approval was obtained from Health Canada and the Austrian Agency for Health and Food Safety. Regulatory approval was not required in Chile. Written informed consent was obtained for all participants and, where applicable, written assent was also obtained. The study protocol was registered on clinicaltrials.gov by JDM on 25/05/2015 (NCT02452762). Results of this pilot trial will not be rolled into the subsequent phase III trial.

### Participating centres

Patients were recruited from the PICU at four tertiary care centres*:* CHEO (Ottawa, Canada), London Health Sciences Centre (London, Canada), Medical University of Graz (Graz, Austria), and Hospital Guillermo Grant Benavente (Concepcion, Chile). Children were also recruited from the Neonatal Intensive Care Unit (NICU) at CHEO.

### Participants and eligibility criteria

Vitamin D deficient children aged > 37 weeks corrected gestational age to < 18 years admitted to ICU with an anticipated ICU stay of > 48 h and who were expected to have access for clinical bloodwork seven days post-intervention were included. VDD for inclusion in this study was defined as a plasma 25(OH)D concentration < 50 nmol/L [27]. This threshold was selected based on our systematic review which showed that a 25(OH)D concentration of < 50 nmol/L was associated with a > two-fold increase in mortality [27]. A list of the study exclusion criteria are presented in Additional file 2. Eligible patients were identified and recruited in the PICU or NICU (CHEO only). In some centers, patients were also identified and pre-consented through the Cardiovascular Surgery Department and randomized if VDD was confirmed at the time of admission to the PICU.

### Randomization

Participants were randomized using a web-based randomization system and assigned a randomization number. A computer-generated randomization list was prepared by the Ottawa Methods Centre at the Ottawa Hospital Research Institute. Patients were randomized 2:1 using random variable block sizes (2–4 patients/block). A 2:1 randomization (high dose: placebo) schema was employed because the control group is not directly pertinent for our primary objective of dose evaluation but required to assess our secondary objectives. Randomization was stratified by patient age (above or below 30 days of age) and by site to account for site-specific practice variation. All study personnel, members of the health care team and patients/families were blinded to study group assignment. To help maintain blinding, the active drug and placebo were identical in appearance, consistency, volume, taste and smell. The randomization number was provided to the site pharmacist and matched to a hard-copy randomization list to determine treatment allocation. The hard-copy randomization list was only accessible to the Ottawa Methods Centre and to the site pharmacist.

### Intervention

The dosing regimen evaluated was identified through a systematic review and meta-regression of pediatric high dose vitamin D trials [51]. Participants randomized to the experimental arm received a cholecalciferol load (Vitamin D3 (Cholecalciferol) Oral Solution 50,000 IU/mL, Euro-Pharm International Canada Inc.) at enrollment at a dose of 10,000 IU/kg (maximum 400,000 IU). Participants randomized to the control group received a placebo solution at enrollment, equivalent in volume to the appropriate dose of cholecalciferol. At the discretion of the health care team (who were blinded to treatment allocation), study participants could also receive routine or standard of care daily vitamin D administration (400–800 IU/day). With the exception of study drug administration (enteral cholecalciferol or placebo), there were no other changes to clinical management and no protocolization of care.

### Research sample collection and analysis

Blood samples were collected either at the time of clinically indicated venipuncture or through existing arterial or central venous line access. If the participant did not have existing lines or planned clinical bloodwork, the study blood sample was not collected. Research blood samples were collected at screening (with additional blood collected at enrollment if the volume from the screening sample was insufficient for all planned analyses), days 1, 2, 3, 7 and hospital discharge for analysis of plasma 25(OH)D and ionized calcium. The research blood samples collected for an ionized calcium concentration were analyzed in real time (unless ionized calcium had been measured through clinical bloodwork in the preceding 24 h). The research blood samples collected to determine plasma 25(OH) concentrations were stored and analyzed in batches by the research laboratory by LC–MS/MS [60]. These samples are referred to throughout the remainder of this manuscript as the *research* samples.

Urine samples were collected at enrollment, on Day 3, 7, and at hospital discharge and analyzed in in real time for calcium:creatinine ratios.

### Safety monitoring

Three serious adverse events (SAEs) were defined a priori that would be considered both unexpected and potentially related to study drug. The events were: (i) gastrointestinal bleeding (requiring blood transfusion) and perforation (requiring surgery) within 48 h of study drug administration; (ii) persistent hypercalcemia (> 24 h in the absence of parenteral calcium administration) with renal failure requiring dialysis, nephrocalcinosis, hemodynamically significant arrhythmia, cardiorespiratory arrest or death; and (iii) new or worsening hypercalciuria with nephrolithiasis, or renal failure leading to dialysis or death. Ionized calcium levels and urine calcium:creatinine ratios from the research samples described above were monitored in real time by the site investigator for persistent hypercalcemia [59] and hypercalciuria (Additional file 4).

In addition, we used the local site clinical laboratory, or the Qualigen FastPak® system, to measure plasma 25(OHD) concentration in real-time from the last blood sample collected prior to hospital discharge (referred to as the *clinical* sample). Three of four participating sites used a radio immunoassay to analyze the clinical samples, and the forth site used LC–MS/MS. The clinical sample result was reviewed by the study nephrologist (PG), who was not involved in clinical care of patients or any other study procedures. Safety procedures were protocolized, and patients with concerns or abnormal research samples were referred to nephrology or endocrinology as outlined in Additional file 4. The frequency of these events by study arm were reviewed by the Data Safety Monitoring Board after 15, 30 and 52 participants reached Day 7 of study enrollment.

### Data collection

Data was collected from the time of study enrollment until 90 days or hospital discharge (whichever occurred first). At enrollment, families completed a questionnaire to help understand their interest in research on VDD in critical illness and inform primary outcome selection for a subsequent Phase III trial. More specifically, families were asked to indicate, other than mortality, the three most important outcomes for a research study evaluating rapid restoration of vitamin D status in critically ill children, and then to indicate all of the outcomes they would consider important.

### Primary outcome

Our primary outcome was the proportion of critically ill children who achieved a plasma 25(OH)D concentration > 75 nmol/L (normalization of vitamin D status) prior to hospital discharge based on the *clinical* samples with imputed concentrations for missing data (see Statistical Analysis for description of imputation methods). We initially intended to perform the primary analysis using the *research* plasma sample collected on Day 7. However, an instrumentation issue recognized during analysis of the batched research samples resulted in inaccurate plasma 25(OH)D measurements. As there was insufficient research sample volume to repeat the laboratory analysis for all research samples, the study steering committee made the decision to evaluate the primary outcome based on the *clinical* sample collected and analyzed in real-time prior to hospital discharge. We have included the research sample results in Additional file 3; the results reported below in the manuscript are based on the clinical sample.

### Secondary outcomes

Our secondary outcomes included the frequency of vitamin D related adverse events and an evaluation of the feasibility of a large phase III trial. Vitamin D related adverse events were defined as: persistent hypercalcemia > 24 h without calcium administration [59]; hypercalciuria as determined by an elevated calcium:creatinine ratio in two sequential post-intervention urine samples; and ultrasound-confirmed nephrocalcinosis. Feasibility outcomes for a multi-centre phase III RCT included protocol adherence, rate of study drop-out, ability to maintain blinding, assessment of the study eligibility criteria, and patient accrual. Criteria for feasibility and proceeding with a Phase III trial were established, and are summarized in Table 4. We also reported baseline values for two potential outcomes for a Phase III trial in order to inform sample size for subsequent phases of this research program: (i) multi-organ dysfunction (measured by the Pediatric Logistic Organ Dysfunction (PELOD-2) score [61]) at enrollment and on Day 3, 7, 30, and PICU discharge; and (ii) PICU length of stay.

### Sample size

The weight-based loading protocol was designed to achieve a 25(OH)D concentration of > 75 nmol/L in 75% of study participants in the intervention arm, with a minimal acceptable proportion of 50% achieving this target. Assuming the true proportion achieving target was 75%, a random sampling of 36 patients would have ~ 90% power to return an estimate in excess of 66% of participants achieving target 25(OH)D. Given an estimate in excess of 66% and a sample size of 36, the lower 95% confidence interval would exclude 50%. To account for an anticipated 5% drop out rate or missing blood samples, we aimed to recruit a total sample size of 60 patients: 40 patients into the high dose arm, and 20 in the placebo arm. Although a control group was not relevant for the primary outcome (evaluating response to vitamin D loading dose), it was determined to be essential for evaluating abnormalities in blood or urine calcium levels. Of these two, urine calcium levels are infrequently measured in the ICU, and having a control group would be essential to interpreting the levels. For example, our pilot randomized controlled trial of pre-operative vitamin D supplementation in stable congential heart disease demonstrated that 17% of patients have elevated urine calcium peri-operatively [62]. Second, without the placebo arm we would not be able to properly evaluate recruitment or our ability to achieve blinding.

### Statistical analysis

All statistical analyses were performed using R version 3.6.3 [63]. The treatment and placebo arms were described separately using means with standard deviations for normally distributed continuous variables or medians with interquartile range values for non-normally distributed variables. Categorical variables were described using frequencies with percentages.

The proportion of participants in the treatment and placebo arm achieving a 25(OH)D concentration > 75 nmol/L by hospital discharge was calculated, with the Wilson’s score used to generate 95% confidence intervals. Additionally, the difference in these proportions between groups was calculated, again with a 95% Wilson confidence interval, and Fisher’s exact test for comparing the proportions. As stated above, due to an instrumentation issue during analysis of the research samples, we instead used the clinical sample analyzed in real-time prior to hospital discharge to evaluate the primary outcome. As a clinical sample for the primary analysis was not available for all patients, an imputation procedure was performed when an accurate research measurement was available. First, using observed pairs of clinical and research measurements of 25(OH)D, a linear regression log-transformed model of clinical sample measurements on log-transformed research sample measurements was fitted. This model was then used to impute the clinical sample measurement when an accurate research sample measurement was available. The results were first reported using imputation of missing 25(OH)D values and second using solely the clinical sample 25(OH)D concentrations. An analysis of covariance (ANCOVA) was performed to compare 25(OH)D concentration from the clinical sample between treatment groups, adjusting for the baseline 25(OH)D concentration at the time of screening (prior to study drug administration).

Secondary analyses included calculating proportions by treatment group of the following potential adverse effects of treatment: presence of nephrocalcinosis, persistent hypercalcemia, and persistent hypercalciuria. Wilson’s score was used to generate 95% confidence intervals for each above-mentioned proportion. As described in the trial protocol paper [59], we also collected and reported common PICU clinical outcomes by treatment group. Statistical tests were not performed to compare the groups.

Sub-analyses were performed whereby the proportion of participants in the treatment arm and in the placebo arm achieving 25(OH)D concentrations > 75 nmol/L was calculated with Wilson’s score’s test to generate 95% confidence intervals within six different subgroupings: (1) patients under and ≥ 40 kg or over; (2) patients with a 25(OH)D concentration at the time of screening above or ≤ 32 nmol/L; (3) newborns (< 30 days old) or ≥ 30 days of age; (4) by country of enrollment, Canada versus outside of Canada; (5) by race, and (6) by admitting diagnosis. To test for subgroup differences a logistic regression model was fit with a main effect of treatment arm, the subgroup variable (e.g. weight category), and an interaction between these two variables. The p-value for the estimated interaction was reported.

### Significant modifications to the study drug protocol

The trial intervention initially involved two doses of study drug (placebo or cholecalciferol): (i) at time of enrollment, and (ii) a day 3 dose dependent on the 25(OH)D concentration achieved. However, after randomization of the first 12 patients, the intervention was adjusted in July 2016 to a single-dose protocol administered at the time of study enrollment. There were multiple reasons for this modification. First, new literature in the adult ICU population demonstrated that administration of a single loading dose of 500,000 IU, comparable to our intervention, raised 25(OH)D concentrations by 80 (± 35) nmol/L [44]. These findings suggested that incorporating a second load into the protocol might be of little value. Secondly, three of the initial twelve patients met the criteria for safety follow-up based on their discharge 25(OH)D concentration. At this time, the threshold for safety follow-up in our study protocol was a 25(OH)D concentration > 150 nmol/L, however, this was later increased to 200 nmol/L based on guidance from the Canadian Paediatric Society [64]. Of these three patients, two had already achieved target 25(OH)D concentrations by Day 3 following a loading single dose. Thirdly, expanding recruitment to additional centres and eventual translation of the dosing regimen would be more feasible with a protocol involving a single loading dose only. Fourth, removing the second loading dose substantially decreased study costs. Finally, since the second loading dose first required determining the patient’s 25(OH)D concentration at Day 3, the chances of accidental unblinding were increased. Of the twelve patients who were randomized while the two-dose protocol was active, seven received > 1 dose of cholecalciferol and their 25(OH)D data was excluded from the analysis. A summary of results for these seven patients is presented in Additional file 5.

## Results

### Screening and recruitment

**Fig. 1:**
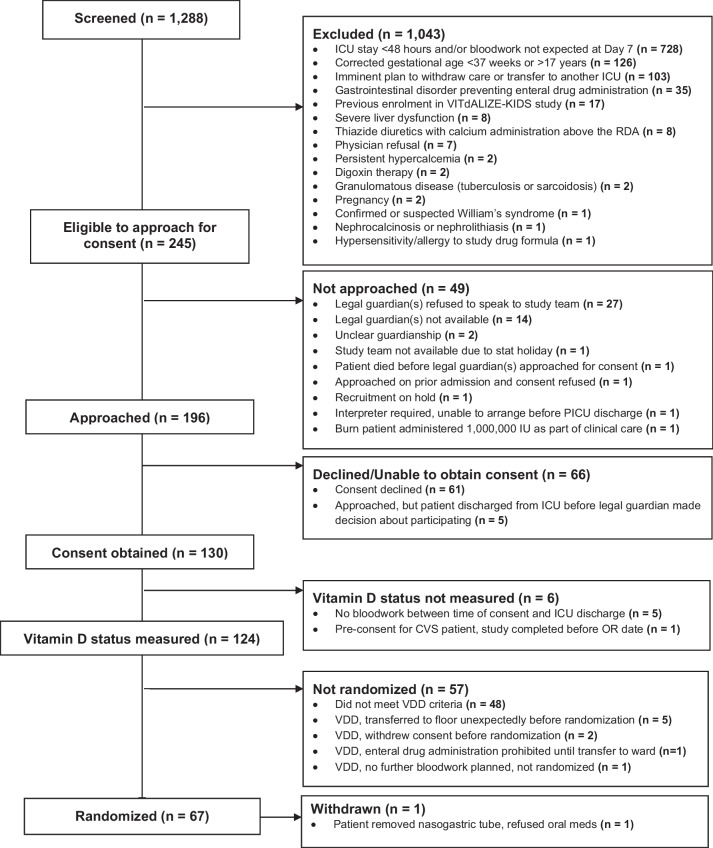
Legend: CONSORT diagram. Abbreviations: ICU: intensive care unit; RDA: recommended dietary allowance; CVS: cardiovascular surgery; OR – Operating room; VDD: vitamin D deficiency; NPO: No per oral

A total of 1,288 children admitted to a participating ICU from January 2016 to August 2017 were screened for eligibility (Fig. 1, Consort Diagram). Of these, 1043 were excluded, most commonly because the clinical team anticipated the patient would have a PICU stay of < 48 h and/or was unlikely to have in-hospital clinical bloodwork on Day 7 (n = 728). Of 245 patients who met clinical eligibility criteria, vitamin D status was measured in 124, and 76 (61%) were VDD. Sixty-seven children were randomized, one of whom was subsequently withdrawn prior to drug administration due to withdrawal of assent. Seven children who were enrolled under the two-dose protocol received > 1 loading dose of cholecalciferol and were excluded from the main analysis. Results for these seven participants are presented in Additional file 5. The main analysis is based on 59 participants: 40 (67.8%) in treatment and 19 (32.2%) in placebo arm. Characteristics of the participants are described in Table 1. The plasma 25(OH)D concentration in each arm at the time of screening (before study-drug administration) was 34.6 nmol/L (IQR: 32.0,41.1) in the treatment arm and 36.5 nmol/L (IQR: 32.8, 41.6) in placebo group. The Pediatric Risk of Mortality (PRISM) III score at PICU admission was 7.0 (IQR: 4.0, 12.0) and 8.0 (IQR: 2.5, 11.5) in the treatment and placebo arms, respectively. In total, 32 (80.0%) and 20 (50.0%) participants in the treatment arm, and 16 (84.2%) and 9 (47.4%) participants in the placebo arm required mechanical ventilation or vasopressor support respectively during PICU admission.

**Table 1:** Demographics by intervention arm

Patient Characteristic	Treatment (*n* = 40)	Placebo (*n* = 19)
**Age (months)**, *median (IQR)*	13.4 (2.1, 86.8)	11.8 (2.1, 87.9)
**Weight (kg)**, *median (IQR)*	12.4 (5.0, 25.0)	7.5 (4.8, 28.1)
**Sex**, *frequency* (%)		
Female	16 (40.0)	9 (47.4)
Male	24 (60.0)	10 (52.6)
**Recruitment country**, *frequency* (%)		
Canada	33 (82.5)	16 (84.2)
Other country	7 (17.5)	3 (15.8)
**ICU type**, *frequency* (%)		
NICU	3 (7.5)	2 (10.5)
PICU	37 (92.5)	17 (89.5)
**Admission season**, *frequency* (%)		
Fall	8 (20.0)	4 (21.1)
Winter	11 (27.5)	7 (36.8)
Spring	9 (22.5)	4 (21.1)
Summer	12 (30.0)	4 (21.1)
**Ethnicity,***frequency (%)*		
Caucasian	17 (42.5)	11 (57.9)
Aboriginal, Inuit	3 (7.5)	1 (5.3)
Black	2 (5.0)	2 (10.5)
Other/Unknown/Missing	18 (45.0)	5 (26.3)
**Chronic condition**^**a**^, *frequency* (%)	15 (37.5)	9 (47.4)
Endocrine condition	1 (6.7)	1 (11.1)
Renal disease	2 (13.3)	0 (0.0)
GI disease	1 (6.7)	1 (11.1)
Malabsorption	1 (6.7)	0 (0.0)
Other	14 (93.3)	7 (77.8)
**Admitting diagnosis**, *frequency* (%)		
Medical	31 (77.5)	12 (63.2)
Cardiac surgery	6 (15.0)	6 (31.6)
General surgery	2 (5.0)	1 (5.3)
Trauma	1 (2.5)	0 (0.0)
Cardiac, non-surgical	0 (0.0)	0 (0.0)
**PRISM III score**, *median (IQR)*	7.0 (4.0, 12.0)	8.0 (2.5, 11.5)
**Mechanical ventilation required**, *frequency* (%)	32 (80.0)	16 (84.2)
**Received catecholamines**, *frequency* (%)	20 (50.0)	9 (47.4)

^a^Please note that conditions are not mutually exclusive and thus will not total 100%

*IQR* Interquartile range, *ICU* Intensive care unit, *PICU* Pediatric intensive care unit, *NICU* Neonatal intenstive care unit, *GI* Gastrointestinal, *PRISM*Pediatric Risk of Mortality

### Primary outcome

**Fig. 2:**
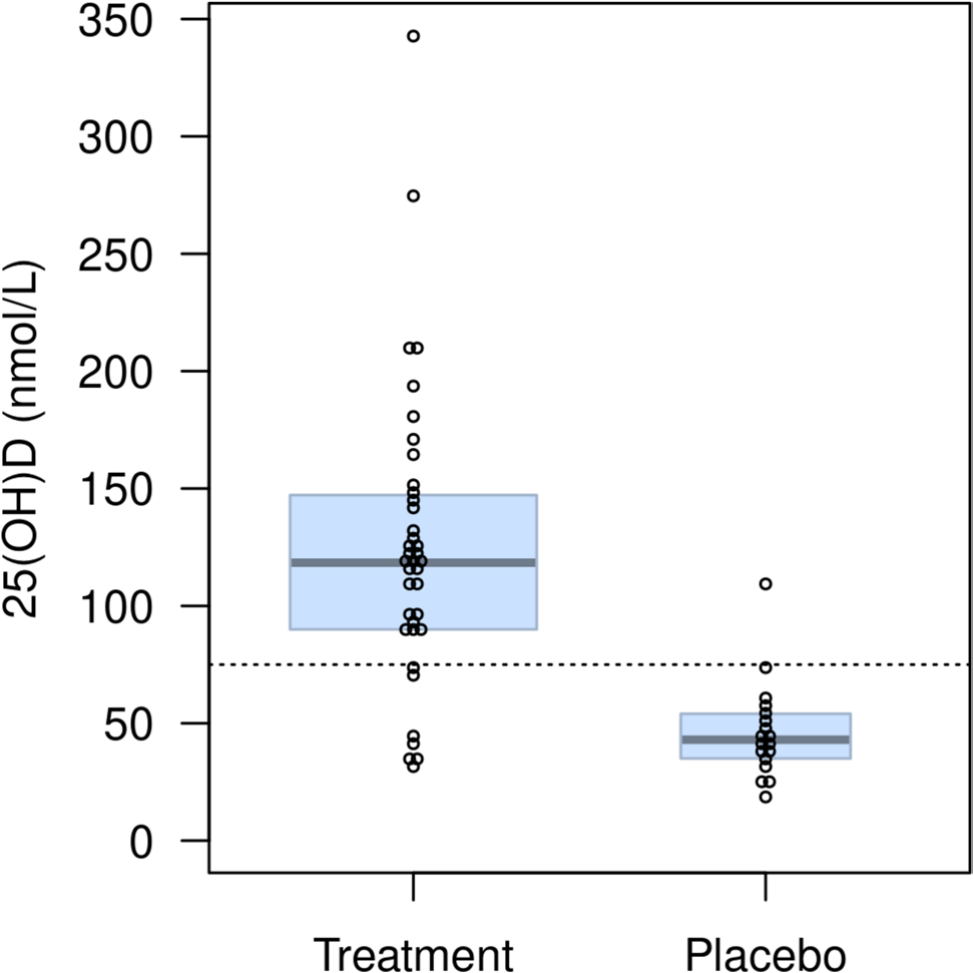
Distribution of plasma 25(OH)D concentration prior to hospital discharge (n = 56). Distribution of plasma 25(OH)D concentration in nmol/L (based on clinical and imputed samples) prior to hospital discharge by treatment arm. In each arm, dots represent individual observations, the box represents the 25^th^ and 75^th^ percentiles and the heavy horizontal line represents the median. Points above the dashed horizontal line at 75 nmol/L represent individuals who have had normalization of vitamin D status

The median study day when the clinical sample was collected and analyzed was 6.5 (5, 10.5) for the treatment arm and 5.5 (4.25, 8) for the placebo arm. A clinical sample was available for 44 patients. Of the 15 patients who did not have a clinical sample collected, 25(OH)D concentrations could be imputed based on an accurate paired research sample for 12 participants. As a result, the primary outcome analysis is based on a total of 56 participants, 38 in the treatment arm and 18 in the placebo arm. The proportion of participants who achieved a plasma 25(OH)D concentration > 75 nmol/L was 81.6% (31/38) (95% CI: 66.6%, 90.8%) in the treatment versus 5.6% (1/18) (95% CI: 1.0%, 25.8%) in the placebo arm (Table 2). This placebo patient did receive routine daily vitamin D supplementation from the health care team (1000–2000 IU) on study days 1 to 7 (total dose over 7 days: 11,000 IU). The estimated difference in proportions was 76% (95% CI: 51%, 86%). The distribution of plasma 25(OH)D concentrations from the clinical samples and imputed samples are illustrated in Fig. 2. As a sensitivity check, the proportion of participants who achieved a plasma 25(OH)D concentration > 75 nmol/L was determined using only the 44 patients who had a clinical sample collected. In the treatment arm, 83.3% (25/30) (95% CI: 66.4%, 92.7%) of participants achieved a 25(OH)D concentration > 75 nmol/L compared with 7.1% (1/14) (95% CI: 1.3%, 31.5%) in the placebo arm (Table 2). The estimated difference in proportions was 76% (95% CI: 47%, 87%). Because the clinical sample was collected prior to hospital discharge instead of Day 7 (as was originally planned), we performed a further sensitivity analysis by determining the proportion of participants who achieved a plasma 25(OH)D concentration > 75 nmol/L based on the Day 7 research sample. In the treatment arm, 27/32 (84.4%) participants had a 25(OH)D concentration > 75 nmol/L at Day 7, compared with 1/11 (9.1%) in the placebo arm.

**Table 2:** Number of patients by treatment arm with a plasma 25(OH)D concentration above 75, 200 and 250 nmol/L

	***N***	**Treatment (*n* = 38)**	**Placebo (*n* = 18)**
**Clinical Sample + Imputed Sample,** plasma *25(OH)D concentration (nmol./L)*^*a*^	56	118.4 (91.0, 146.3)	43.0 (35.5, 53.0)
**Clinical Sample + Imputed Sample,***frequency* (%)^a^			
> 75 nmol/L	56	31 (81.6)	1 (5.6)
> 200 nmol/L	56	4 (10.5)	0 (0.0)
> 250 nmol/L	56	2 (5.3)	0 (0.0)
	**N**	**Treatment (*n* = 30)**	**Placebo (*n* = 14)**
**Clinical Sample Only*** plasma 25(OH)D concentration (nmol./L)*^b^	44	118.0 (94.2, 146.1)	43.0 (36.0, 54.5)
**Clinical Sample Only,***frequency* (%)^b^			
> 75 nmol/L	44	25 (83.3)	1 (7.1)
> 200 nmol/L	44	3 (10.0)	0 (0.0)
> 250 nmol/L	44	1 (3.3)	0 (0.0)

^a^Based on Clinical Sample with imputed results for missing blood samples (primary outcome data)

^b^Based on Clinical Sample only (no imputed results)

25(OH)D – 25-hydroxyvitamin D

Further, subanalysis sought to explore relationships between post-treatment vitamin D levels and other participant characteristics. No significant relationship between weight, age, initial screening plasma 25(OH)D concentration, country of recruitment, race, admitting diagnosis, or vasopressor administration on the proportion of participations who achieved 25(OH)D concentration > 75 nmol/L. The results of the subgroup analysis are presented in Additional file 6. Given small numbers it was not possible to perform a statistical analysis on the relationship between baseline gastro-intenstinal disease and post-drug vitamin D levels. Post-drug 25(OH)D levels of 211 nmol/l and 74 nmol/l were achieved in the two treatment arm participants identified with significant reflex (+ gastrostomy tube) and failure to thrive due to malabsorption, respectively. As no participants vomited within one hour of drug administration, no related analysis was possible.

### Plasma 25(OH)D concentrations

An ANCOVA was performed to examine for an association between the final level of log transformed 25(OH)D and treatment group adjusting for the baseline log transformed plasma 25(OH)D concentration. The p-value for an interaction between baseline level and group was p = 0.2. The interaction was removed and the model was refitted. The model estimate, accounting for screening 25(OH)D level, was that, on average, the final plasma 25(OH)D concentrations were 2.66 times higher (95% CI: 1.98, 3.56) in the treatment group compared to the placebo group. Tables providing details regarding the ANOVA analyses are presented in Additional file 7.

### Hypervitaminosis and Vitamin D related adverse events

The number of patients in each arm with a clinical 25(OH)D concentration > 75 nmol/L, 200 nmol/L and 250 nmol/L (including imputed value) are presented in Table 2. In total, four patients in the treatment arm achieved a 25(OH)D concentration > 200 nmol/L, with two of these > 250 nmol/L. Ionized calcium and urine calcium:creatinine concentrations are presented in Table 3. No patients developed persistent hypercalcemia following study drug administration. In total, 6/40 (15.0%) (95% CI 7.1%, 29.1%) in the treatment arm developed hypercalciuria in two sequential post-drug urine samples following study drug adminstration, compared to 4/19 (21.1%) (95% CI 8.5%, 43.3%) patients in the placebo arm. Overall, 37/59 (62.7%) of participants had an abdominal ultrasound performed following study drug administration; 26/40 (65.0%) patients in the treatment arm and 11/19 (57.9%) patients in the placebo arm. No participants were found to have nephrocalcinosis on ultrasound among 40 receiving treatment and one who received placebo. There were no adverse events or serious adverse events related or potentially related to study participation.

**Table 3:** Ionized calcium concentration and urine calcium:creatinine ratios by treatment arm at enrollment, Day 3, Day 7 and at hospital discharge

**Time Point**	**N**	**Treatment Arm (*n* = 40)**			**Placebo Arm (*n* = 19)**	
		**Lowest Ionized Calcium Concentration (mmol/L)** **Median (IQR)**	**Highest Ionized Calcium Concentration (mmol/L) Median (IQR)**	**N**	**Lowest Ionized Calcium Concentration (mmol/L)** **Median (IQR)**	**Highest Ionized Calcium Concentration (mmol/L)** **Median (IQR)**
**Day 0**	25	1.17 (1.13, 1.22)	1.22 (1.17, 1.28)	11	1.18 (1.14, 1.23)	1.21 (1.17, 1.29)
**Day 3**	22	1.16 (1.06, 1.26)	1.21 (1.15, 1.29)	8	1.12 (1.09, 1.18)	1.27 (1.14, 1.27)
**Day 7**	20	1.17 (1.12, 1.23)	1.22 (1.18, 1.27)	7	1.17 (1.10, 1.19)	1.18 (1.17, 1.30)
**DSC**	21	1.24 (1.18, 1.32)	1.26 (1.18, 1.32)	13	1.20 (1.15, 1.27)	1.24 (1.18, 1.29)
**Time Point**	**N**	**Urine Calcium:Creatinine Ratio (mol/mol) Median (IQR)**		**N**	**Urine Calcium:Creatinine Ratio (mol/mol) Median (IQR)**	
**Day 0**	31	1.22 (0.24, 2.45)		14	0.92 (0.44, 2.40)	
**Day 3**	30	1.08 (0.34, 2.08)		16	1.20 (0.23, 2.69)	
**Day 7**	27	0.99 (0.50, 1.42)		10	0.55 (0.20, 2.28)	
**DSC**	15	0.92 (0.35, 1.04)		4	1.90 (0.86, 3.42)	

*IQR* Interquartile range, *DSC* Discharge

### Feasibility outcomes

The feasibility criteria for proceeding with a phase III trial, along with the outcome from this trial, and whether or not each criteria was met are summarized in Table 4. There were no major protocol violations related to the study drug or safety procedures, which met our criteria to establish the feasiblity of protocol adherence of major study drug administration or safety procedures deviations occuring in < 20% of enrolled patients. One participant, during the period with the two dose regimen, was unblinded at the request of the principal investigator after the clinical team ordered a 25(OH)D concentration 3 weeks following receipt of study drug and it returned at 229 nmol/L. This result was discussed with the study safety officer who confirmed the study plasma 25(OHD) concentration measured on day 8 was 100 nmol/L. The patient was unblinded confirming the patient was in the treatment arm and indicating the patient had received a second half-dose load of cholecalciferol on Day 3. The patient was followed as an outpatient, and at a clinical visit ~ 4 months later had a normal abdominal ultrasound and a 25(OH)D level of 76 nmol/L. One patient was withdrawn from the study following randomization, but prior to study drug administration. Although the patient was initially not asked for assent given their critial illness, the patient regained capacity and then refused all oral drug administration. No patients withdrew from the study following study drug administration. The patient accrural rate of 3.4 patients/month exceeded our feasibility requirement of 2.8 patients/month. A Day 7 blood sample was collected for 56/67 (84%) of enrolled patients.

**Table 4:** Assessment of Phase III trial feasibility

Feasibility Criteria		Outcome	Feasibility Criteria Met
Study Withdrawal	Withdrawal rate of < 10% of enrolled patients will be considered successful	One patient was randomized but withdrawn prior to study drug administration due to withdrawal of assent	Yes
Patient Accrual	An accrual rate of 67 patients within 2 years (average 2.8 patients/month) will be considered successful	67 patients enrolled over a 20-month period (average 3.4 patients/month). This is equivalent to 1.9 patients/site/month	Yes
Ability to Maintain Blinding	Care team or pharmacy request for unblinding in < 10% of enrolled patients will be considered successful	Blinding was broken for one study patient (1% of enrolled patients) at the request of the principal investigator. No patients were unblinded at the request of pharmacy or the clinical team	Yes
Eligibility Criteria^a^	Ability to obtain a blood sample in 95% of enrolled patients will be considered successful	A Day 7 blood sample was collected for 56 patients (84% of enrolled patients)	No
Protocol Adherence	Occurrence of major protocol deviations with regard to study drug administration or safety procedures in < 20% of enrolled patients will be considered successful	No deviations related to study drug administration or safety procedures	Yes

^a^Eligibility criteria was evaluated based on our ability to predict ICU stay longer than 48 h and bloodwork access at 7 days for collection of blood sample at Day 7

### Routine vitamin D supplementation prescribed at the discretion of the medical care team

Fifteen (37.5%) and 9 (47.4%) of patients in the treatment arm and placebo arm respectively received daily vitamin D supplementation as part of routine care at the discretion of the medical care team during the first 7 days following study drug admnistraiton. The majority (11/15, 73%) of patients in the treatment arm received daily supplementation on all 7 days, ranging from 400–1000 IU/day. Just under half (4/9, 44%) of patients in the placebo arm received a daily dose of vitamin D supplementation on each of the seven days following study drug administration, ranging from 400–1000 IU/day.

### Phase III trial outcome assessment

Forty families completed the outcome selection questionnaire. Of these 29 (72.5%) believed that having normal vitamin D status could be important to recovery, and indicated they would be concerned if their child was determined to have lower than desired levels of vitamin D during their hospital stay. Thirty-four (85.0%) agreed researchers should determine how to correct VDD and whether it helps children recover from critical illness. The top three outcomes, and all the outcomes indicated as important, are summarized in Additional file 8. The outcome measures most commonly selected as a “top three” outcome were: (i) your child's overall quality of life and functioning after hospital discharge, (ii) time it takes for your child's organs (heart, lungs, kidneys) to function normally, and (iii) your child's pain level after hospital discharge.

Five patients died in hospital, three in the treatment arm and two in the placebo arm. The median (IQR) PELOD-2 score at the time of study enrollment was 4.0 (1.0, 6.0). There were 42 participants still in the PICU on Day 3. The median (IQR) Day 3 PELOD-2 score, corresponding to the 48–72 h time period required for majority conversion of cholecalciferol to 25(OH)D, was 5.0 (2.0, 7.0). On Day 7, representing a time period where maximal 25(OH)D levels would have been achieved and maintained for multiple days, the median (IQR) PELOD-2 score was 3.5 (1.8, 5.2) for the 28 participants still admitted to the PICU. The median (IQR) PELOD-2 score at PICU discharge was 1.0 (0.0, 2.0). The median (IQR) PICU length of stay for the study cohort was 8.9 (6.0,13.8) days. Additional clinical outcomes are summarized in Table 5 and in Additional file 9.

**Table 5:** Clinical outcomes by treatment arm

Outcome	Treatment (*n* = 40)	Placebo (*n* = 19)
**In hospital mortality**, *frequency* (%)	3 (7.5)	2 (10.5)
**Mechanical Ventilation (hrs)**, median (IQR)	149.2 (75.2, 247.2)	172.2 (86.0, 333.1)
**NIV (days)**, median (IQR)	3.9 (0.7, 8.9)	0.5 (0.2, 4.4)
**PICU length of stay (days)**, median (IQR)	8.7 (6.4, 12.7)	9.0 (6.0, 14.4)
**Hospital length of stay (days)**, median (IQR)	16.2 (9.4, 30.9)	15.8 (9.3, 31.2)
**Nosocomial infection**, *frequency* (%)	7 (17.5)	2 (10.5)

*NIV* Non-invasive ventilation, *PICU* Pediatric intensive care unit

## Discussion

This pilot phase II RCT confirms that a single 10,000 IU/kg dose can rapidly and safely normalize plasma 25(OH)D concentrations in critically ill children identified as vitamin D deficient (< 50 nmol/L) during admission to the PICU, and that proceeding with a Phase III trial is feasible.

We recruited vitamin D deficient (median ~ 35 nmol/L) children from 4 PICUs and 1 NICU at 4 academic centers in Canada, Austria and Chile. The evaluation of baseline characteristics demonstrates the study cohort was severely ill, with 80% requiring mechanical ventilation and 50% receiving vasoactive agents. The loading dose regimen rapidly increased blood levels within 1 day, peaked on days 2 and 3, with 80% of participants exceeding the target blood threshold of 75 nmol/L. These findings are similar to studies with comparable doses administered to critically ill adults with admission 25(OH)D levels < 50 nmol/L. For example, the VITdAL study reported an increase of 50 nmol/L following a 540,000 IU cholecalciferol dose, with 52% exceeding 75 nmol/L [45]. Similarly, in the VIOLET trial, an adult acute lower respiratory tract infection population at high risk for acute respiratory distress syndrome and ICU admission increased blood 25(OH)D concentrations from an average of 28 to 117 nmol/L (75% exceeding 75 nmol/L) with a 540,000 IU enteral dose [65]. No pediatric trials have evaluated a similar age or weight base dosing regimen on critically ill children. The most related study evaluated the 10,000 IU/kg dose recommended from our systematic review, in a cohort of VDD children undergoing cardiac surgery for congenital heart disease (Tetralogy of Fallot) [66]. Cholecalciferol dosing approximately two weeks prior to surgery produced significantly higher concentrations in the treatment arm relative to control (83.5 vs. 27.4 nmol/L) immediately before the surgery. Given that blood 25(OH)D concentrations peaks ~ 72 h following loading dose administration and then begins to decline [51], and the known half-life of vitamin D, blood 25(OH)D concentrations may have been higher if they had been measured closer to loading dose administration.

In addition, this study also assessed the feasibility of the weight-based loading dose regimen through an evaluation of vitamin D toxicity data. Loading dose cholecalciferol, when using excessive or repeated doses or in particular in infants and young children, has been linked to hypercalcemia, nephrocalcinosis and renal failure [67]. Critically ill patients may be at greater risk for adverse events due severe organ dysfunction, higher prevalence of genetic abnormalities, and potential interactions with common ICU medications [68, 69]. However, we did not document any cases of persistent hypercalcemia, consistent with the low hypercalcemia rate (2.6%) observed in a meta-analysis of pediatric interventional trials [7]. These findings align with the hypercalcemia rates reported in the adult VITdAL-ICU (≤ 1%) and VIOLET (≤ 3%) trials, with no differences between control and intervention arms [45, 65]. Similarly, in a pediatric cardiac surgery trialblood calcium levels in the arm receiving a 10,000 IU/kg pre-operative dose were not elevated compared to the control group [66]. Evaluation of urine calcium and nephrocalcinosis data in our trial failed to identify reason for concern with hypercalciuria rates similar between treatment (15%) and usual care (21%). Nonetheless, the baseline rate was considerably higher than the pooled 2.5% rate reported in the systematic review of pediatric vitamin D clinical trials (no critically ill children) [51]. This difference was not unexpected in the pediatric critical care setting due to the significant presence of systematic inflammation, renal dysfunction, and medication use (e.g. diuretics). Again, findings were very similar to those presented in the adult VITdAL-ICU trial, where both arms had 25% hypercalciuria rates both before and following a 540,000 IU cholecalciferol load [45]. Sahu and colleagues also reported similar or potentially lower average post cardiac surgery calcium:creatine ratios in the cohort receiving the cholecalciferol load (2.0 vs 1.1, p = 0.16) [66]. All of this suggests that hypercalciuria is not an appropriate biomarker of excess vitamin D levels in the ICU setting. Finally, further suggesting the safety of this regimen, was the absence of any definitive cases of nephrocalcinosis in the treatment arm or serious adverse events potentially related to vitamin D.

An important study observation relates to significant variability in post loading dose 25(OH)D concentrations. Eight of 10 of participants in the treatment arm exceed the 75 nmol/L threshold following the single loading dose. However, while the group average 25(OH)D concentration achieved was successful, individual patient responses were variable with some patients exhibiting minimal change and not reaching concentrations > 75 nmol/L (n = 7). In addition, a few patients achieved concentrations > 200 nmol/L (n = 4) or > 250 nmol/L (n = 2). Significant variability in post-study drug concentrations has been previously recognized, with regression analysis of data from pediatric interventional trial literature calculating the SD to average 42% of the mean 25(OH)D [51]. The calculated SD of ~ 63 nmol/L on a mean plasma 25(OH)D concentration of ~ 126 nmol/L and the observation ~ 20% of study participants had concentrations either below 50 nmol/L or above 200 nmol/L is consistent with these previous findings. These findings are also in line with the post-loading dose SD of 52 nmol/L (day 7) and 58 nmol/L (day 3) in the VITdAL and VIOLET trials, respectively [45, 65]. Both VITdAL (24%) and VIOLET (12%) also reported a significant number of non-responders, defined as post-drug 25(OH)D concentrations below 50 nmol/L. Comparing results with respect to elevated 25(OH)D is more challenging due to application of different thresholds across studies. In the VITdAL analyses, Amrein et al. applied a 150 nmol/L threshold, reporting 13% of study participants above this level with the two highest 25(OH)D concentrations in their treatment arm as ~ 265 nmol/L [45], below the 375 nmol/L threshold where risk of acute toxicity may begin to rise [70, 71]. With respect to the VIOLET trial, the upper target 25(OH)D concentration was set as 300 nmol/L, with only one patient exceeding that value [65]. The two highest 25(OH)D measurements in our pilot study were 343 and 275 nmol/L, and with no symptoms of vitamin D toxicity. Our protocol indicated a change in dose would be considered if > 10% of the participants receiving the loading dose achieved a 25(OH)D concentration above 250 nmol/L [59], and this was not exceeded [70, 71]. As the first evaluation of this dosing regimen in a high-risk population, we made the decision to use a conservative threshold of 250 nmol/L. Although our aim was to avoid 25(OH)D concentrations > 250 nmol/L, we still recommend proceeding with this dosing regimen for a subsequent large-scale trial. Vitamin D toxicity is time-dependent, and transient levels > 200 nmol/L do not appear relevant [71]. Importantly, there were no cases of persistent hypercalcemia, clinically significant hypercalciuria, nephrocalcinosis or any other adverse events related to vitamin D supplementation observed in this pilot study. Further, reducing the dose would increase the number of patients who do not achieve post-supplementation 25(OH)D concentrations > 75 nmol/L and dilute the impact of the loading dose regimen on clinical outcome in a Phase III trial. This decision was also informed by observations by our group, and others that 25(OH)D concentrations peak 72 h following loading dose administration and then rapidly begin to fall [51], with an average decline of 10 nmol/L week observed in our systematic review of pediatric loading dose trials [51]. For example, Thacher et al*.* found that 25(OH)D3 concentrations fell by 53% and 59% in rachitic and healthy children 14 days following administration of a loading dose of 50,000 IU [72].

The results of this phase II trial support the feasibility of large-scale, multicentre phase III trial powered for clinical outcome. We met our a priori established feasibility criteria for protocol adherence, blinding, study withdrawal and patient accrual. The patient accrual rate was 3.4 patients per month (~ 1.9 patients/month/site). However, with the exception of the lead site, the other sites were only recruiting for ≤ 5 months, while peak recruitment in critical care RCTs is generally not achieved until at least 7 months after a site initiates recruitment [73]. Therefore, we believe that a reasonable expected accrual rate for a multi-year, multi-site RCT would be 2 patients/month/site, which is within the range observed in other completed large, multi-centre PICU RCTs performed in the last decade by large research consortiums [74–81]. Of note, this expected accrual rate is dependent on a VDD prevalence consistent with that observed during this pilot study. Our ability to collect blood samples at Day 7 was slightly lower than anticipated (84% vs > 95%). In this trial, the Day 7 blood sample was essential to establish the efficacy of the dosing regimen, and to evaluate for toxicity; however, sample collection would be less important for a more pragmatic Phase III trial focused on clinical outcome. Given that 25(OH)D concentrations peak ~ 72 h following loading dose administration, adjusting the protocol to allow sample collection anytime between Day 2 to Day 7 would increase the frequency of sample collection, as clinical bloodwork frequency tends to be higher earlier during a PICU admission. This would allow a future Phase III trial to also perform a metabolomic sub-study to supplement the existing adult literature [82–84].

Recognizing the importance of patient-centred outcomes in clinical trials, we engaged with participating families to seek input on the primary outcome for the Phase III trial. Feedback from families participating in this trial and a concurrent survey by Merrit et al. [85], indicates that health-related quality of life is the most important outcome for families in a Phase III clinical trial in critically ill children [85]. Historically, PICU RCTs have used hospital-based outcomes (e.g. length of stay in PICU, hospital mortality) that allow primary outcome data collection for 100% of enrolled patients. In contrast, previous observational studies evaluating health-related quality of life in PICU patients at ≥ 1 month have reported completion rates of 52 to 79% [86–88]. Using a primary outcome that will be collected following hospital discharge for the Phase III RCT means that some participants will be lost to follow-up, and this should be accounted for during sample size calculation. Further, strategies to maximize follow-up will be essential, such as collecting multiple points of contact information, including locators; pre-notifying participants of an upcoming follow-up visit; making additional attempts to contact if the first attempt is not successful; monitoring loss to follow up to identify concerns early; and the addition of incentives for completing questionnaires [89–91].

## Conclusion

A single 10,000 IU/kg dose can rapidly and safely normalize plasma 25(OH)D concentrations in critically ill children with VDD during admission to the PICU, but with significant variability in post loading dose 25(OH)D concentrations. We established that proceeding with a Phase III multicentre trial to evaluate the impact of this loading dose regimen on clinical outcomes is feasible. Importantly, using a patient oriented primary outcome determined after hospital discharge such as health-related quality of life will require thoughtful implementation of strategies to minimize loss to follow-up. Based on the results of this pilot study, we launched the Phase III trial in June 2019 (NCT03742505), and as of February 2023, have enrolled 263 patients across 9 participating Canadian PICUs.

### Supplementary Information

**Additional file 1.** CONSORT 2010 checklist of information to include when reporting a pilot or feasibility trial*.

**Additional file 2.** Inclusion and Exclusion Criteria.

**Additional file 3.** Research Sample 25(OH)D Results.

**Additional file 4.** Definition of vitamin D related adverse events monitored in real-time through lab values from the clinical laboratory, and safety procedures for elevated lab values in the VITdAL-PICU Pilot Study.

**Additional file 5.** Summary of patients who received two doses of study drug and were excluded from the study analysis.

**Additional file 6.** Sub analysis Results. Supplemental Table 6. Proportion achieving the primary outcome by weight, age, screening level, country, admitting diagnosis and race subgroups.

**Additional file 7.** ANCOVA multivariable models a) including an interaction term and b) without the interaction term.

**Additional file 8.** Results of the Outcome Measure Selection Questionnaire.

**Additional file 9.** Additional Clinical Outcomes.

## Data Availability

The datasets used and/or analysed during the current study are available from the corresponding author on reasonable request.

## abbreviations

25(OH)D: 25-Hydroxyvitamin D
CCCTG: Canadian Critical Care Trials Group
CHEO: Children’s Hospital of Eastern Ontario
CONSORT: Consolidated Standards of Reporting Trials
CPB: Cardiopulmonary bypass
ECMO: Extracorporeal membrane oxygenation
IU: International units
NICU: Neonatal intensive care unit
PELOD: Pediatric Logistic Organ Dysfunction Score
PICU: Pediatric intensive care unit
PRISM: Pediatric Risk of Mortality Score
RCT: Randomized controlled trial
VDD: Vitamin D deficiency
