# Supplementary material for: A phase II dose evaluation pilot feasibility randomized controlled trial of cholecalciferol in critically ill children with vitamin D deficiency (VITdAL-PICU study)
Source: BMC Pediatr. 2023 Aug 14;23:397. doi: 10.1186/s12887-023-04205-9 (PMC10424361; doi:10.1186/s12887-023-04205-9)
Supplement: Supplementary file 2 — Additional file 2. Inclusion and Exclusion Criteria. [file 12887_2023_4205_MOESM2_ESM.pdf]

Additional File 2: Inclusion and Exclusion Criteria

Inclusion Criteria

1. Admitted to ICU
2. Corrected gestational age >37 weeks to age < 18 years
3. Expected ICU admission in excess of 48 hours
4. Likely to have access for bloodwork at 7 days of hospital admission (clinical bloodwork or lines)

Exclusion Criteria

1. Significant gastrointestinal disorder preventing enteral drug administration
2. Hypercalcemia (excluding transient abnormalities and those related to parenteral calcium administration for hypocalcemia)
3. Confirmed or suspected William's syndrome
4. Patient known to have nephrocalcinosis or nephrolithiasis
5. Imminent plan for withdrawal of care or transfer to another ICU
6. Physician refusal
7. Previous enrolment into the study
8. Patient known to have granulomatous disease (tuberculosis or sarcoidosis)
9. Severe liver dysfunction/failure
10. Patient known to have hypersensitivity or allergy to vitamin D or any of the non-medicinal ingredients of the formulation
11. Patient on thiazide diurectics who is also receiving regular ongoing calcium supplementation above the daily recommended intake (for reasons other than hypocalcemia)
12. Adolescent female of child-bearing age with a positive pregnancy serum test
13. Patient on digoxin-therapy
