# Supplementary material for: A phase II dose evaluation pilot feasibility randomized controlled trial of cholecalciferol in critically ill children with vitamin D deficiency (VITdAL-PICU study)
Source: BMC Pediatr. 2023 Aug 14;23:397. doi: 10.1186/s12887-023-04205-9 (PMC10424361; doi:10.1186/s12887-023-04205-9)
Supplement: Supplementary file 3 — Additional file 3. Research Sample 25(OH)D Results. [file 12887_2023_4205_MOESM3_ESM.pdf]

Additional File 3: Research Sample 25(OH)D Results

Plasma 25(OH)D concentrations in nmol/L among those enrolled in Canada

Timepoint	Treatment (n=33)		Placebo (n=16)	
	n	median (IQR) nmol/L	*n*	median (IQR) nmol/L
**Screening**	33	37.9 (32.0, 42.0)	16	36.5 (34.6, 41.5)
**Enrollment**	16	54.9 (42.8, 71.7)	8	38.0 (33.5, 55.9)
**Day 1**	23	160.6 (106.8, 215.0)	13	40.3 (33.8, 48.6)
**Day 2**	22	198.0 (113.8, 265.1)	13	47.2 (37.2, 58.6)
**Day 3**	25	176.1 (127.7, 249.8)	12	44.0 (36.7, 67.4)
**Day 7**	26	198.9 (128.8, 226.5)	8	44.8 (42.5, 65.4)
**Hospital Discharge**	17	161.0 (156.8, 224.5)	4	39.4 (36.4, 49.1)

Plasma 25(OH)D concentrations in nmol/L among those enrolled outside of Canada

Timepoint	Treatment (*n*=7)		Placebo (*n*=3)	
	n	median (IQR) nmol/L	*n*	median (IQR) nmol/L
**Screening**	7	20.2 (20.1, 27.0)	3	27.5 (26.9, 34.6)
**Enrollment**	1	73.5	1	63.6
**Day 1**	6	112.8 (74.8, 134.4)	3	36.6 (33.0, 43.2)
**Day 2**	5	220.7 (181.4, 256.5)	2	38.0 (33.2, 42.9)
**Day 3**	5	241.4 (164.7, 386.4)	2	39.6 (35.9, 43.3)
**Day 7**	6	182.7 (70.5, 319.6)	3	39.6 (36.2, 46.4)
**Hospital Discharge**	1	297.2	0	NA (NA, NA)
