# Supplementary material for: A phase II dose evaluation pilot feasibility randomized controlled trial of cholecalciferol in critically ill children with vitamin D deficiency (VITdAL-PICU study)
Source: BMC Pediatr. 2023 Aug 14;23:397. doi: 10.1186/s12887-023-04205-9 (PMC10424361; doi:10.1186/s12887-023-04205-9)
Supplement: Supplementary file 4 — Additional file 4. Definition of vitamin D related adverse events monitored in real-time through lab values from the clinical laboratory, and safety procedures for elevated lab values in the VITdAL-PICU Pilot Study. [file 12887_2023_4205_MOESM4_ESM.pdf]

Additional File 4: Definition of vitamin D related adverse events monitored in real-time through lab values from the clinical laboratory, and safety procedures for elevated lab values in the VITdAL-PICU Pilot Study

Adverse Event	Study Thresholds	Definition	Safety Procedures
**Hypercalcemia**	Ionized calcium level:<br>>1.40 mmol/L<br>>1.45 mmol/L for children <8 weeks of age	Persistent hypercalcemia for >24 hours in absence of calcium administration	Endocrinology consult, managed clinically as determined by Endocrinology
**Hypercalciuria (calcium:creatinine ratio)**	Age-based thresholds:<br><1 2.2 mol/mol<br>1-2 1.5 mol/mol<br>2-3 1.4 mol/mol<br>3-5 1.1 mol/mol<br>5-7 0.8 mol/mol<br>7-17 0.7 mol/mol	Hypercalciuria, as determined by a calcium:creatinine ratio above the study threshold in two sequential urine samples (excluding enrolment sample).	Nephrology consult, case reviewed to determine need for repeat urine sample, abdominal ultrasound and/or clinical management by Nephrology
**Hypervitaminosis D**	Plasma 25(OH)D concentration >200 nmol/L	Plasma 25(OH)D concentration >200 nmol/L in Day 7 sample	Endocrinology consult, managed clinically as determined by Endocrinology

From: McNally D, Amrein K, O'Hearn K, Fergusson D, Geier P, Henderson M, Khamessan A, Lawson ML, McIntyre L, Redpath S, Weiler HA, Menon K; Canadian Critical Care Trials Group. Study protocol for a phase II dose evaluation randomized controlled trial of cholecalciferol in critically ill children with vitamin D deficiency (VITdAL-PICU study). Pilot Feasibility Stud. 2017 Dec 8;3:70. doi: 10.1186/s40814-017-0214-z. PMID: 29234503; PMCID: PMC5721544.
