# Supplementary material for: A phase II dose evaluation pilot feasibility randomized controlled trial of cholecalciferol in critically ill children with vitamin D deficiency (VITdAL-PICU study)
Source: BMC Pediatr. 2023 Aug 14;23:397. doi: 10.1186/s12887-023-04205-9 (PMC10424361; doi:10.1186/s12887-023-04205-9)
Supplement: Supplementary file 5 — Additional file 5. Summary of patients who received two doses of study drug and were excluded from the study analysis. [file 12887_2023_4205_MOESM5_ESM.pdf]

Additional File 5: Summary of patients who received two doses of study drug and were excluded from the study analysis

Characteristic	Participant						
	**01-001**	**01-061**	**01-062**	**01-063**	**01-066**	**01-069**	**01-070**
**Age (mo.)**	0.2	176.2	1.1	13.3	1.8	147.9	3.1
**Weight (kg)**	3.8	195.0	4.9	9.4	11.1	54.6	16.2
**Sex**	Male	Male	Male	Male	Male	Female	Male
**Admission season**	Winter	Winter	Winter	Winter	Spring	Spring	Spring
**Diagnosis**	Surgical, Cardiac	Medical	Medical	Surgical, Cardiac	Medical	Medical	Medical
**Mechanical ventilation[a]**	Yes	No	No	Yes	No	Yes	Yes
**PICU length of stay (hours)**	212	151	179	354	184	189	289
**Survival Status at PICU Discharge**	Alive	Alive	Alive	Alive	Alive	Alive	Alive
**Received catecholamines**	Yes	No	No	Yes	No	Yes	Yes
**Group Assignment**	High Dose	Placebo	High Dose	High Dose	Placebo	High Dose	Placebo
**Day 3 plasma 25(OH)D concentration, nmol/L (CHEO)**	61.1	64.0	158.1	149.6	<32.0[c]	59.1	55.8
**Number of doses received[b]**	1.5	1.5	1	1	2	1.5	1.5
**Day 7 25OHD, nmol/L (Research)**	143.6	31.5	306.6	192.0	38.6	109.9	54.0
**Hospital Discharge plasma 25OHD concentration, nmol/L (Clinical Sample)**	99.5	73.3	163.5	129.3	25.0	75.0	37.0

[a]Defined as endotracheal intubation

[b]Based on Day 3 25OHD level; <50 nmol/L = 10000 IU/KG to a maximum of 400000IU; 50-75 nmol/L = 5000 IU/KG to a maximum of 400000IU; > 75 nmol/L = placebo is given

[c]Lower threshold for point of care device is 32.0 nmol/L. Values lower than 32.0 nmol/L reported as <32.0 nmol/L.

12 patients randomized to 2 dose regimen; 7 patients received 2 doses
