# Supplementary material for: A phase II dose evaluation pilot feasibility randomized controlled trial of cholecalciferol in critically ill children with vitamin D deficiency (VITdAL-PICU study)
Source: BMC Pediatr. 2023 Aug 14;23:397. doi: 10.1186/s12887-023-04205-9 (PMC10424361; doi:10.1186/s12887-023-04205-9)
Supplement: Supplementary file 6 — Additional file 6. Sub analysis Results. Supplemental Table 6. Proportion achieving the primary outcome by weight, age, screening level, country, admitting diagnosis and race subgroups. [file 12887_2023_4205_MOESM6_ESM.pdf]

Additional File 6: Sub analysis Results

Supplemental Table 6: Proportion achieving the primary outcome by weight, age, screening level, country, admitting diagnosis and race subgroups

Category	Treatment group	Placebo group	Difference in proportion (95% CI)	p-value*
Weight <40 kg (n=48)	27/33 (81.8%)	1/15 (6.7%)	75% (95% CI 47%, 86%)	0.86
Weight >= 40 kg (n=8)	4/5 (80.0%)	0/3 (0.0%)	80% (95% CI 10%, 96%)	
Age <1 month (n=9)	3/6 (50.0%)	0/3 (0.0%)	50% (95% CI -14%, 81%)	0.24
Age >= 1 month (n=47)	28/32 (87.5%)	1/15 (6.7%)	81% (95% CI 53%, 90%)	
Screening plasma 25(OH)D concentration ≤32 nmol/L (n=20)[a]	13/16 (81.2%)	0/4 (0.0%)	81% (95% CI 31%, 93%)	0.83
Screening plasma 25(OH)D concentration > 32 nmol/L (n=36)	18/22 (81.8%)	1/14 (7.1%)	75% (95% CI 43%, 87%)	
From Canada (n=46)	26/31 (83.9%)	1/15 (6.7%)	77% (95% CI 49%, 88%)	0.78
Outside Canada (n=10)	5/7 (71.4%)	0/3 (0.0%)	71% (95% CI 5%, 92%)	
Medical diagnosis (n=40)	24/29 (82.8%)	1/11 (9.1%)	74% (95% CI 40%, 86%)	0.852
Other diagnosis (n=16)	7/9 (77.8%)	0/7 (0.0%)	78% (95% CI 30%, 94%)	
Race, including Caucasian (n=28)	15/17 (88.2%)	1/11 (9.1%)	79% (95% CI 43%, 90%)	0.88
Race including other (n=28)	16/21 (76.2%)	0/7 (0.0%)	76% (95% CI 35%, 89%)	

[a]32.0 nmol/L was chosen as the cutoff as this represented the lowest level of detection of the screening assay used in the Canadian centres.
*p-value from logistic regression model to test for subgroup differences.

Subanalysis by Weight
There were 48 participants who were under 40kg and had a 25(OH)D measurement. Of these, there were 33 in the treatment arm with a median (IQR) plasma 25(OH)D concentration of 118.0 (90.0, 147.2) nmol/L. There were 15 in the placebo arm with a median (IQR) plasma 25(OH)D concentration of 44.0 (38.1, 52.0) nmol/L. In the treatment arm, 27/33 (81.8%) achieved a plasma 25(OH)D concentration of >75 nmol/L compared with 1/15 (6.7%) in the placebo arm. The estimated difference in proportions with 95% confidence intervals is 75% (95% CI: 47%, 86%).

Among the 8 participants 40 kg or more with a 25(OH)D level, 5 were in the treatment arm. The median plasma 25(OH)D concentration in the treatment arm was 123.0 (95.0, 124.0) nmol/L versus 26.0 (26.0, 41.0) nmol/L in the placebo arm. In the treatment arm, 4/5 (80.0%) achieved

a plasma 25(OH)D concentration >75 nmol/L, versus 0/3 (0.0%) in the placebo arm. The estimated difference in proportions with 95% confidence intervals is 80% (95% CI: 10%, 96%).

Subanalysis by age

There were 9 participants who were under one month of age. Among these, there were 6 in the treatment arm, and the median (IQR) plasma 25(OH)D concentration was 80.7 (37.9, 121.8) nmol/L. Three were in the placebo arm, and the median (IQR) plasma 25(OH)D plasma was 44.0 (42.0, 46.4) nmol/L. In the treatment arm, 3/6 (50.0%) achieved a plasma 25(OH)D concentration >75 nmol/L compared with 0/3 (0.0%) in the placebo arm. The estimated difference in proportions with 95% confidence intervals is 50% (95% CI: -14%, 81%).

Among those one month of age or older, there were 32 in the treatment arm with a 25(OH)D, and the median (IQR) plasma 25(OH)D concentration was 120.9 (94.8, 156.2) nmol/L. Fifteen in the placebo arm with a 25(OH)D, and the median (IQR) plasma 25(OH)D concentration was 42.0 (33.7, 55.0) nmol/L. In the treatment arm, 28/32 (87.5%) achieved a 25(OH)D plasma concentration >75 nmol/L compared with 1/15 (6.7%) in the placebo arm. The estimated difference in proportions with 95% confidence intervals is 81% (95% CI: 53%, 90%).

Subanalysis by Screening 25(OH)D Concentration (≤32 nmol/L)

There were 20 participants who had a screening plasma 25(OH)D concentration ≤32 nmol/L. Among those, there were 16 in the treatment group, and the median (IQR) plasma 25(OH)D concentration was 125.9 (93.7, 169.4) nmol/L. The remaining four participants were in the placebo group, and the median (IQR) plasma 25(OH)D concentration was 33.7 (28.6, 35.5) nmol/L. In the treatment group, 13/16 (81.2%) participants achieved a plasma 25(OH)D concentration >75 nmol/L and 0/4 (0.0%) in the placebo group. The estimated difference in proportions is 81% (95% CI: 31%, 93%).

There were 36 participants who had a plasma 25(OH)D concentration at screening above 32 nmol/L. Among these, there were 22 patients in the treatment group, and the median (IQR) plasma 25(OH)D concentration was 117.0 (91.0, 127.5) nmol/L and there were 14 in the placebo group, and the median (IQR) plasma 25(OH)D concentration was 47.4 (40.5, 55.5) nmol/L. In the treatment group, 18/22 (81.8%) participants achieved a plasma 25(OH)D concentration >75 nmol/L compared with 1/14 (7.1%) in the placebo group. The estimated difference in proportions is 75% (95% CI: 43%, 87%).

Subanalysis by Country

There were 46 participants who were recruited in Canada and had a clinical sample 25(OH)D concentration. Among those, 31 were in the treatment group and the median (IQR) plasma 25(OH)D concentration was 118.0 (94.5, 145.1) nmol/L. The remaining 15 were in the placebo group, and the median (IQR) plasma 25(OH)D concentration was 44.0 (37.0, 55.0) nmol/L. In the treatment group, 26/31 (83.9%) participants achieved a plasma 25(OH)D concentration >75 nmol/L compared with 1/15 (6.7%) in the placebo group. The estimated difference in proportions was 77% (95% CI: 49%, 88%). Ten participants were recruited outside of Canada. Among those recruited from outside of Canada, there were 7 in the treatment group, and the median (IQR) plasma 25(OH)D concentration was 133.0 (66.7, 161.5) nmol/L. Three were in the placebo group, and the median (IQR) plasma 25(OH)D concentration was 37.2 (34.8, 41.6) nmol/L. In the treatment group, 5/7 (71.4%) participants achieved a plasma 25(OH)D concentration >75 nmol/L compared with 0/3 (0.0%) in the placebo group. The estimated difference in proportions was 71% (95% CI: 5%, 92%).

Subanalysis by Admitting diagnosis

There were 40 participants who were admitted for a medical diagnosis and had a clinical sample 25(OH)D concentration. Among those, 29 were in the treatment group and the median (IQR) plasma 25(OH)D concentration was 118.0 (90.0, 133.0) nmol/L. The remaining 11 were in the placebo group, and the median (IQR) plasma 25(OH)D concentration was 42.0 (29.2, 53.0) nmol/L.

In the treatment group, 24/29 (82.8%) participants with a medical admission diagnosis achieved a plasma 25(OH)D concentration >75 nmol/L compared with 1/11 (9.1%)in the placebo group. The estimated difference in proportions was 74% (95% CI 40%, 86%).

There were 16 participants who were admitted for a non-medical diagnosis and had a clinical sample 25(OH)D concentration. Among those, 9 were in the treatment group and the median (IQR) plasma 25(OH)D concentration was 153.0 (110.0, 193.0) nmol/L. The remaining 7 were in the placebo group, and the median (IQR) plasma 25(OH)D concentration was 44.0 (38.1, 51.4) nmol/L. In the treatment group, 7/9 (77.8%) with the surgical diagnosis achieved a plasma 25(OH)D concentration >75 nmol/L compared with 0/7 (0.0%) in the placebo group. The estimated difference in proportions was 78% (95% CI 30%, 94%).

Subanalysis by Race

There were 28 participants who were Caucasian and had a clinical sample 25(OH)D concentration. Among those, 17 were in the treatment group and the median (IQR) plasma 25(OH)D concentration was 118.8 (94.0, 153.0) nmol/L. The remaining 11 were in the placebo group, and the median (IQR) plasma 25(OH)D concentration was 46.0 (39.5, 58.5) nmol/L. In the treatment group, 15/17 (88.2%) participants achieved a plasma 25(OH)D concentration >75 nmol/L compared with 1/11 (9.1%) in the placebo group. The estimated difference in proportions was 79% (95% CI 43%, 90%).

There were 28 participants who were of either black/aboriginal/inuit/other race and had a clinical sample 25(OH)D concentration. Among those, 21 were in the treatment group and the median (IQR) plasma 25(OH)D concentration was 118.0 (90.0, 143.0) nmol/L. The remaining 7 were in the placebo group, and the median (IQR) plasma 25(OH)D concentration was 35.0 (29.2, 45.4) nmol/L. In the treatment group, 16/21 (76.2%) participants achieved a plasma 25(OH)D concentration >75 nmol/L compared with 0/7 (0.0%) in the placebo group. The estimated difference in proportions was 76% (95% CI 35%, 89%).

Subanalysis by vasopressor use

Among the 40 participants in the treatment group who received a loading dose of cholecalciferol, 20 (50%) received vasopressors and 20 (50%) did not. Of 19 available vitamin D levels in the vasopressor group, 15/19 (79%) participants achieved a plasma 25(OH)D concentration >75 nmol/L compared to 16/19 (84%) participants in the no vasopressor group. A Fisher's exact test comparing proportions found no statistical difference between the groups (p=1.0).
