# Supplementary material for: A phase II dose evaluation pilot feasibility randomized controlled trial of cholecalciferol in critically ill children with vitamin D deficiency (VITdAL-PICU study)
Source: BMC Pediatr. 2023 Aug 14;23:397. doi: 10.1186/s12887-023-04205-9 (PMC10424361; doi:10.1186/s12887-023-04205-9)
Supplement: Supplementary file 7 — Additional file 7. ANCOVA multivariable models a) including an interaction term and b) without the interaction term. [file 12887_2023_4205_MOESM7_ESM.pdf]

Additional File 7: ANCOVA multivariable models a) including an interaction term and b) without the interaction term.

A)

Characteristic	Beta	95% CI[1]	p-value
Log(25-OH-D baseline level)	1.1	-0.28, 2.6	0.11
Treatment Group			
Placebo	—	—	
Cholecalciferol loading dose group	4.5	-1.0, 10	0.11
log(25-OH-D baseline level) * Cholecalciferol loading dose group	-0.99	-2.5, 0.55	0.2

B)

Characteristic	Beta	95% CI[1]	p-value
Log(25-OH-D baseline level)	0.29	-0.23, 0.81	0.3
Treatment group			
Placebo	—	—	
Cholecalciferol loading dose group	0.98	0.68, 1.3	<0.001

[1]CI = Confidence Interval.

Footnote: In order to represent the treatment effect on the scale of 25-OH-D it is necessary to exponentiate the beta coefficient above as well as the confidence interval. The result is that the 25-OH-D is estimated to be 2.66 times higher (95% CI: 1.98, 3.56) in the treatment group compared to the placebo group.
