# Supplementary material for: A phase II dose evaluation pilot feasibility randomized controlled trial of cholecalciferol in critically ill children with vitamin D deficiency (VITdAL-PICU study)
Source: BMC Pediatr. 2023 Aug 14;23:397. doi: 10.1186/s12887-023-04205-9 (PMC10424361; doi:10.1186/s12887-023-04205-9)
Supplement: Supplementary file 8 — Additional file 8. Results of the Outcome Measure Selection Questionnaire. [file 12887_2023_4205_MOESM8_ESM.pdf]

Additional File 8: Results of the Outcome Measure Selection Questionnaire

Part A: Top Three Outcomes Selected by Forty Families for a Randomized Controlled Trial of Rapid Restoration of Vitamin D Status in Critically Ill Children (n = 40). Families were provided with a list of potential outcomes, as well as an option to specify “Other”, and asked to indicate, other than mortality, the top three outcomes that they would consider important for a research study evaluating rapid restoration of vitamin D status in critically ill children.

**Outcome Measure**	**N (%)**
Your child's overall quality of life and functioning after hospital discharge	30 (75.0%)
Time it takes for your child's organs (heart, lungs, kidneys) to function normally	27 (67.5%)
Your child's pain level after hospital discharge	17 (42.5%)
Length of stay in the intensive care unit and hospital	16 (40.0%)
Your child's ability to return to school and daily activities	9 (22.5%)
Your child's intelligence after hospital discharge	6 (15.0%),
Effect of the illness on rest of your family	4 (10.0%)
Time taken off work for caregiver(s)	3 (7.5%)
Financial cost of the illness (to the hospital and government)	2 (5.0%)
Financial cost of the illness (for your family)	1 (2.5%)
Other[a]	4 (10.0%)
No answer selected*	2 (5.0%)

[a]Other Outcomes indicated by families included: Efficiency of raising vitamin D levels, Readmission to hospital, Overall Recovery Time, and finding answers to child's issues

Part B: All Outcomes Selected as Important by Forty Families for a Randomized Controlled Trial of Rapid Restoration of Vitamin D Status in Critically Ill Children (n = 40). Families were provided with a list of potential outcomes, as well as an option to specify "Other", and asked to indicate, other than mortality, all of the outcomes that they would consider important for a research study evaluating rapid restoration of vitamin D status in critically ill children.

**Outcome Measure**	**N (%)**
Your child's pain level after hospital discharge	31 (77.5%)
Your child's overall quality of life and functioning after hospital discharge	30 (75.0%)
Time it takes for your child's organs (heart, lungs, kidneys) to function normally	30 (75.0%)
Length of stay in the intensive care unit and hospital	26 (65.0%)
Your child's intelligence after hospital discharge	24 (60.0%)
Effect of the illness on rest of your family	21 (52.5%)
Your child's ability to return to school and daily activities	20 (50.0%)
Time taken off work for caregiver(s)	15 (37.5%)
Financial cost of the illness (for your family)	15 (37.5%)
Financial cost of the illness (to the hospital and government)	8 (20.0%)
Other[a]	5 (12.5%)
No answer selected*	5 (12.5%)

[a]Other Outcomes indicated by families included: Length of stay after ICU, Overall recovery time, Complications due to illness, Finding answers, and Readmission to hospital
