# Supplementary material for: A phase II dose evaluation pilot feasibility randomized controlled trial of cholecalciferol in critically ill children with vitamin D deficiency (VITdAL-PICU study)
Source: BMC Pediatr. 2023 Aug 14;23:397. doi: 10.1186/s12887-023-04205-9 (PMC10424361; doi:10.1186/s12887-023-04205-9)
Supplement: Supplementary file 9 — Additional file 9. Additional Clinical Outcomes. [file 12887_2023_4205_MOESM9_ESM.pdf]

Additional File 9: Additional Clinical Outcomes

Outcome	Treatment (*n*=40)	Placebo (*n*=19)
**Hypocalcemia in the PICU**, *frequency* (%)	16 (40.0)	7 (36.8)
**Total Fluid Intake (ml) Enrollment**, median (IQR)	974.7 (630.0, 1686.5)	909.0 (470.2, 1636.9)
**Total Fluid Intake (ml) Day 1**, median (IQR)	1074.0 (580.2, 1543.8)	925.3 (521.7, 1452.9)
**Total Fluid Intake (ml) Day 2**, median (IQR)	1139.4 (575.1, 1508.4)	932.0 (494.8, 1411.7)
**Received PRBCs**, *frequency* (%)	12 (30.0)	8 (42.1)
**Received FFP**, *frequency* (%)	3 (7.5)	2 (10.5)
**Received platelets**, *frequency* (%)	7 (17.5)	5 (26.3)
**Received dialysis**, *frequency* (%)	3 (7.5)	1 (5.3)
**Received CRRT**, *frequency* (%)	1 (2.5)	2 (10.5)
